# The role of contrast-enhanced ultrasound (CEUS) in the evaluation of scrotal trauma: a review

**DOI:** 10.1186/s13244-020-00874-7

**Published:** 2020-05-19

**Authors:** Gibran T. Yusuf, Vasileios Rafailidis, Stephen Moore, Benjamin Hawthorn, Cheng Fang, Dean Y. Huang, Maria E. Sellars, Paul S. Sidhu

**Affiliations:** 1 Department of Radiology, King’s College Hospital NHS Foundation Trust, London, UK; 2 Department of Radiology, St Georges Hospital NHS Foundation Trust, London, UK

**Keywords:** Contrast-enhanced ultrasound, Testicular trauma, Testis-sparing surgery, Scrotal trauma, Haematoma

## Abstract

Testicular trauma is common, usually trivial and rarely requires hospital attendance, but if it does, then imaging becomes essential as scrotal assessment may be difficult due to pain and/or scrotal disruption. Ultrasound (US) assumes a crucial role as other cross-sectional modalities have a limited use in the acute presentation. Despite the acceptable accuracy of conventional US techniques, there are limitations which hinder a thorough evaluation, critically the assessment of tissue viability, crucial for clinical management and prognosis. Contrast-enhanced ultrasound (CEUS) has been shown to offer improved flow visualisation and tissue perfusion compared with conventional Doppler techniques. CEUS can accurately and confidently demonstrate the viability of testicular parenchyma, delineate fracture lines and haematomas and guide treatment for testis-sparing surgery or orchidectomy. The purpose of this review is to present the literature, familiarise physicians with the principles of CEUS and findings of scrotal trauma and illustrate the main abnormalities through characteristic and educative cases.

## Key points

- CEUS is a portable, cost-effective, quick and safe tool which can assist with planning of testis-sparing surgery and overall treatment planning of patients sustaining scrotal trauma.
- CEUS is more sensitive than colour Doppler US in visualising tissue vascularity, establishing tissue viability and avoiding unnecessary emergency surgery or orchidectomy.
- CEUS is valuable in diagnosing sequelae of testicular trauma such as intra-testicular haematomas, fractures, rupture, ischaemia and extra-testicular haematoma.

## Background

Scrotal injury is common but rarely results in hospital presentation and makes up 1% of all trauma, most commonly occurring in the first three decades of life [1–4]. Most cases result from blunt force trauma and are usually self-limiting [1–4]. Hospital admissions related to scrotal trauma are usually from patients with penetrating or severe blunt trauma. In these patients, evaluation of testicular viability is essential. Emergency surgery may be required for testicular salvage, only feasible in the acute setting. Alternatively, when the testis is not viable, orchidectomy may be required when there is clear non-viability. However, orchidectomy should be avoided if possible, as morbidity issues include diminished fertility and body dysmorphia [3].

Clinical assessment following scrotal trauma is often limited due to scrotal pain and swelling. Therefore, imaging is the cornerstone for diagnosing traumatic scrotal injury and guiding the treatment plan between conservative versus operative management. Cross-sectional imaging techniques (computed tomography and magnetic resonance imaging) are either impractical or inconclusive in the setting of scrotal trauma [3, 4]. Ultrasound (US) is ideal for assessing the testis given the superficial location and is considered the gold standard in both the acute and non-acute setting [3, 5]. Colour Doppler ultrasound (CDUS) provides further information on tissue vascularity but it can be equivocal or unreliable in acute cases as it may not detect low flow states, e.g. in paediatric patients [6, 7].

Contrast-enhanced ultrasound (CEUS) has gained increasing popularity worldwide as an imaging technique, particularly following the Food and Drug Administration (FDA) approval in paediatric and adult use in focal liver lesions [8]. The ability to provide dynamic vascular imaging in real time without radiation exposure or iodinated contrast has led to increasing utility of ultrasound contrast agents (UCA’s) in a series of different applications. Official guidelines from the European Federation of Societies for Ultrasound in Medicine and Biology (EFSUMB) describe the usefulness of CEUS in many areas [9, 10]. The most widely recognised and well-established use of CEUS is the characterisation of focal liver lesions [10], although there is growing evidence regarding the value and accuracy of many applications including renal, testicular or endocavitary. When it comes to blunt abdominal trauma, CEUS has a promising diagnostic accuracy for detection of solid organ injuries and is deemed beneficial for hemodynamically stable patients sustaining isolated blunt moderate-energy abdominal trauma [11, 12]. Ultrasound contrast agents (most commonly Lumason/SonoVue^TM^, Bracco, Italy) used in CEUS examination are non-nephrotoxic, the phospholipid shell being metabolised by the liver and the sulphur hexafluoride gas component being exhaled through the lungs. The technique can be performed in patients with renal impairment and needs no prior laboratory testing. Moreover, UCA have a lower risk of adverse reactions compared to the contrast agents currently used in computed tomography (CT) and magnetic resonance imaging (MRI) [13–15]. CEUS is also cost-effective, quick, portable and repeatable safely with multiple injections, all without radiation exposure [10–13]. In the testis, the most important application is in assessing the presence or absence of vascularity in a number of conditions, including spermatic cord torsion, epididymo-orchitis, segmental infarction and neoplasia [2, 6, 16–18].

CEUS has the potential to become an indispensable tool in the assessment of acute scrotal trauma, providing significantly improved levels of accurate and diagnostic imaging [1, 19], helping increase clinician confidence. This is particularly pertinent for surgical decision regarding whether or not to undertake emergency surgery and importantly whether the affected testicle is salvageable [1]. With increased diagnostic information as well as advances in patient care, CEUS could preclude the need for exploratory surgery in the setting of acute scrotal trauma.

## Main text

### Physics of CEUS

CEUS is most commonly performed using an UCA consisting of 3–5 μm sized microbubbles of sulphur hexafluoride gas encapsulated in an outer phospholipid shell (SonoVue) [20, 21]. Due to their size, microbubbles can travel through the pulmonary circulation, reaching every vessel in the body and remain within the blood pool being unable to traverse the endothelium, representing a purely intravascular contrast agent [6, 7, 13, 21]. When microbubbles are exposed to the US beam, they undergo a non-linear oscillation, producing harmonic frequencies, received by the transducer. Optimal oscillation is achieved at a resonant frequency of 3–5 MHz, a range of frequencies commonly used for abdominal applications. The harmonic frequencies generated by the microbubbles are used to separate these from the echoes linearly reflected by static tissue, and thus the scanner can selectively visualise the UCA and display contrast-specific images. This separation is achieved with the so-called pulse-inversion technique, currently available in many US devices. This technique is similar to digital subtraction angiography and produces real-time images of tissue perfusion with an excellent spatial and temporal resolution. CEUS provides information both on a macro-vascular level (visualising large vessels such as the carotids) but also on a micro-vascular level, being able to visualise capillaries. In the setting of scrotal imaging where a linear high-frequency transducer is used, the range of sizes of the microbubbles that can resonant at the higher frequency is less, with the bell-shaped curve of microbubble sizes, and most resonate best at 3–5 MHz. A higher mechanical index (MI) applied also results in earlier disruption of microbubbles. A higher dose of 4.8 ml of the UCA should be used for achieving optimal enhancement of testicular parenchyma [9, 22].

#### Scrotal CEUS technique

The technique for CEUS of the acute scrotum needs to be adjusted to preserve maximum patient comfort and diagnostic quality [2]. The patient should be supine and holding his penis towards his abdomen, while a towel is used to support and stabilise the testis. Liberal amounts of gel should be applied prior to scanning the affected area.

Initial B-mode US is undertaken in the conventional manner, and high-frequency linear transducer ranging between 7 and 15 MHz is used. The examination of the testis should include checking the integrity of tunica albuginea and paratesticular tissues such as the epididymis, scrotal wall and the presence of fluid. B-mode US should be always complemented with colour or power Doppler US in order to assess tissue vascularity, a finding relevant to establish tissue viability.

For CEUS examination, the patient will require intravenous access for contrast administration. It is often helpful to undertake the CEUS examination with a split screen approach, with B-mode and CEUS images displayed sided by sided, allowing low MI and lower quality B-mode image to localise the area of interest. An alternative is to have a contrast-specific image overlaid on a low MI B-mode image. These techniques may not be necessary for global abnormalities such a complete testicular infarction. While CEUS has an excellent safety profile [15, 13], anaphylactic reactions have been documented hence, prompt access to resuscitation equipment should be available [12, 14].

#### Normal scrotal CEUS appearances

**Fig. 1:**
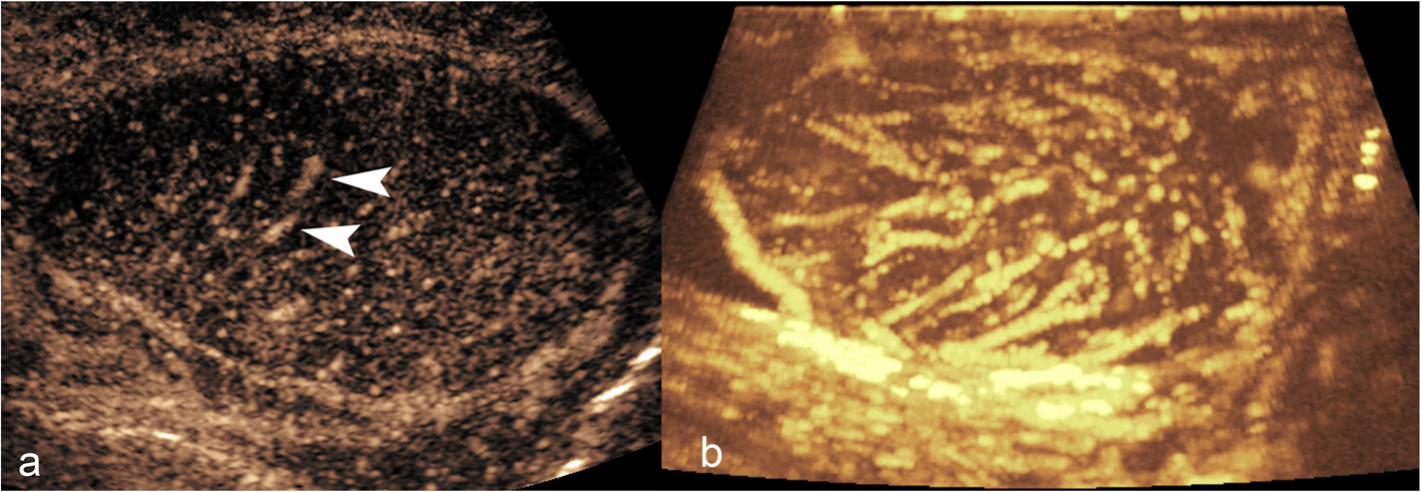
Normal CEUS appearances of testicular parenchyma. Static image (**a**) showing the normal linear/striated mediastinal vessels outlined by microbubbles (arrowheads). Temporal prolonged contrast acquisition image (MIP) (**b**) showing the vascular architecture of normal testicular parenchyma

Normal testicular parenchyma is homogenous with an echogenic surface line indicating the tunica albuginea. On CEUS, the testis should enhance homogeneously with a striated pattern (Fig. 1) representing UCA within the normal intratesticular vascular anatomy. To help demonstrate uniform uptake throughout the testis, the UCA can be accumulated within an image over a period of time to provide a maximum intensity projection image (MIP) for better vascular visualisation (Fig. 1). In the setting of trauma, this technique can be used to definitively determine areas of avascular parenchyma.

### Findings of testicular trauma

#### Intratesticular Haematoma

Intratesticular haematoma is an uncommon finding in scrotal trauma, although the true incidence may be underreported as patient may be asymptomatic. A variety of ultrasound findings are seen depending on when the scan is performed in relation to when the traumatic event occurred [23]. Acute haematomas may appear isoechoic, only causing heterogeneity within the testis, whereas more chronic organising haematomas have hypoechoic, complex solid cystic appearances which critically decrease in size on follow-up scans to eventual resolution [6, 23]. They are usually peripheral, sub-tunical in location, but central haematomas may also be seen when there has been penetration trauma, such as during sperm retrieval [6].

**Fig. 2:**
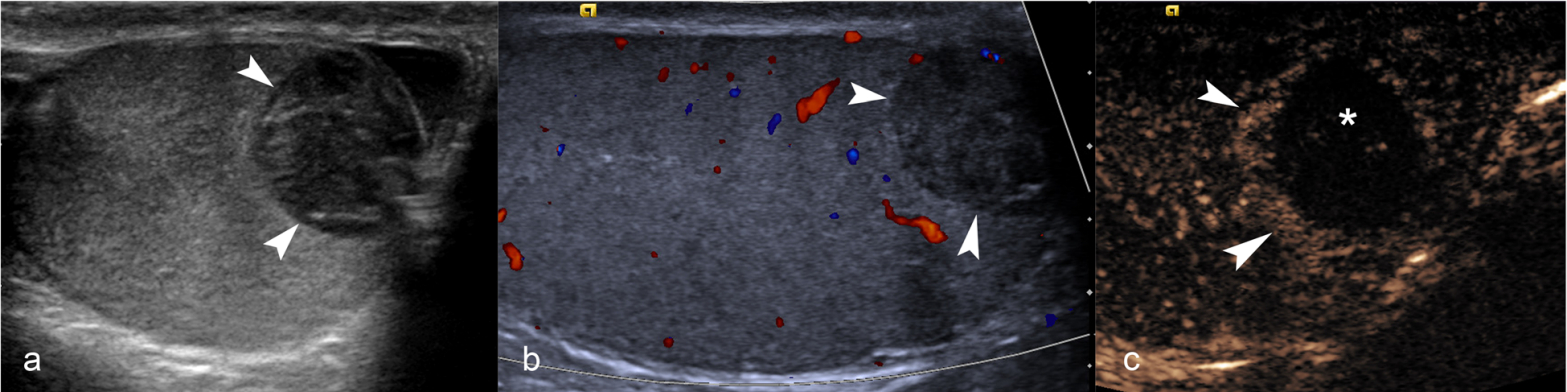
CEUS appearances of intratesticular haematoma after blunt trauma. B-mode image (**a**) showing an intratesticular rounded hypoechoic lesion (arrowheads), associated with hydrocele. On colour Doppler US (**b**), the lesion (arrowheads) showed no internal blood flow signals. CEUS (**c**) confirmed the absence of internal vascularity (asterisk), then unlikely a tumour and in keeping with that of haematoma. Note the peri-lesional hyperaemia (arrowheads) and the presence of two internal echoes, representing artefact from echogenic content

**Fig. 3:**
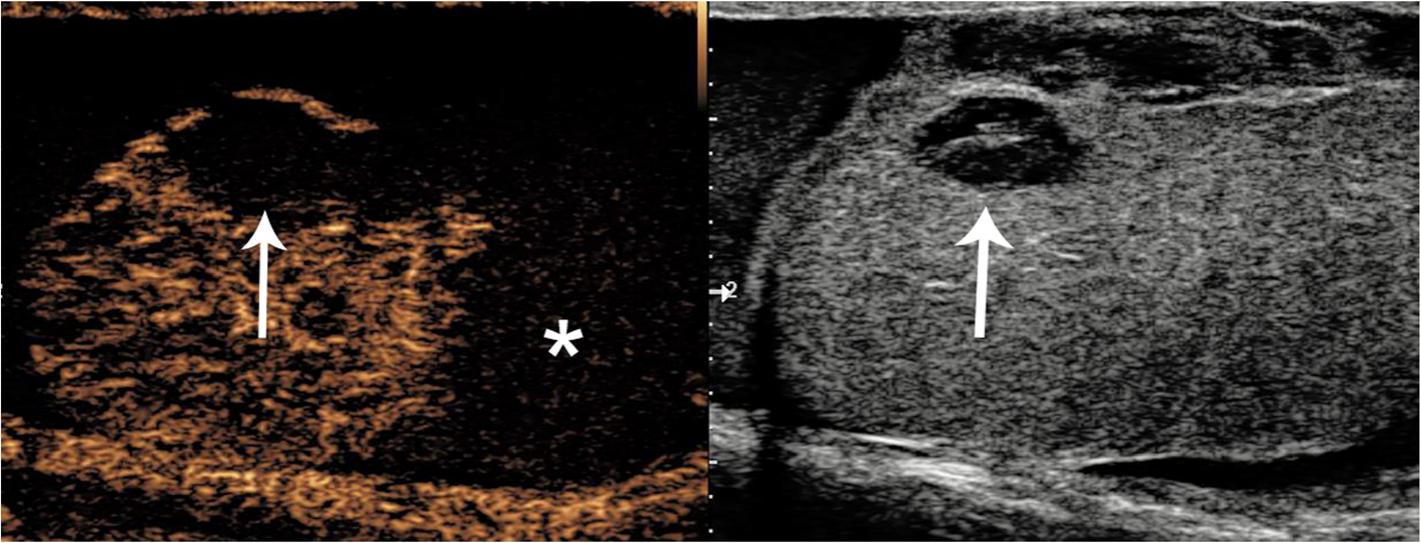
CEUS appearances of haematoma and ischaemia after blunt trauma. Dual-display image showing contrast-specific (left) and low MI B-mode image (right). A rounded hypoechoic and well-circumscribed lesion is seen (arrow), which shows no enhancement, in keeping with an intratesticular haematoma. Importantly, the lower pole of the testis appears heterogeneous on B-mode and shows no enhancement on CEUS, indicating the diagnosis of ischaemia (asterisk). The changes of the area of ischaemia are subtle on the B-mode image alone. Note the ability of CEUS to clearly define the borders of infarction which may be obscure on B-mode imaging alone

**Fig. 4:**
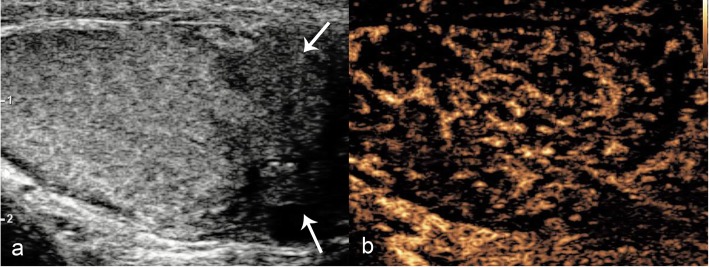
CEUS excluding ischaemia. B-mode (**a**) image demonstrated a hypoechoic, atrophic and heterogeneous lower pole (arrows) raising suspicion of post-surgical infarction. CEUS (**b**) demonstrated homogeneous perfusion of the entire testis, thus excluding the diagnosis and precluding the need for further surgical intervention

A history of trauma may be misleading, and it is important to differentiate intratesticular haematoma from incidentally detected neoplasms [6] which are found in up to 10–20% of scrotal trauma. The presence of intra-lesional vascularity on CDUS and lack of progressive resolution on follow-up imaging should raise suspicion of neoplasm [6]. CEUS can reliably determine the presence or absence of intralesional vascularity helping to exclude neoplastic lesions, ultimately preventing an orchidectomy [1, 6, 7]. Intratesticular haematomas appear on CEUS (Figs. 2 and 3) as well-defined, non-enhancing lesions. In some cases, enhancing septations may be appreciated which were not demonstrable on CDUS, and mild perilesional hyper-enhancement may be seen [6]. The use of a UCA will help differentiate true size of the haematoma and highlight areas of secondary ischaemia [1] which would expedite surgical management [6]. Conversely**,** the visualisation of homogenous enhancement of the entire testis on CEUS negates the need for further investigation (Fig. 4).

#### Acute testicular rupture

Testicular rupture is a rare condition requiring extensive force from a blunt or penetrating injury resulting in loss of integrity of the tunica albuginea. This requires urgent urological referral as 80% of testes can be salvaged if diagnosis is made within 72 h of injury, after which there is a high rate of orchidectomy [1, 23, 24].

**Fig. 5:**
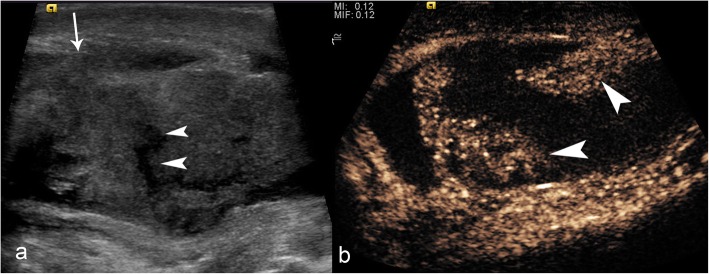
Conventional US and CEUS findings of testicular rupture. On B-mode (**a**), a hypoechoic linear disruption of the testicular parenchyma is demonstrated (arrowheads). Note the discontinuation of tunica albuginea with protrusion of echogenic material, indicating rupture (arrow). CEUS (**b**) determined the amount of viable parenchyma (arrowheads) which aided in pre-operative decision-making and allowed for the salvaging of viable testicular tissue avoiding the need for orchidectomy

The B-mode appearances of a testicular rupture are usually diagnostic with sensitivity and specificity at 100% and 93.5%, respectively [23]. The definitive finding in testicular rupture is discontinuity of the hyperechoic tunica albuginea surrounding the testis. Further findings include an associated distortion of the testicular contour, parenchymal heterogeneity and haematocoele, and in combination, these findings improve sonographic sensitivity [1, 24, 25]. There is almost inevitable loss of the tunica vasculosa resulting in ischaemia of at least a portion of the testis, which would necessitate debridement if the testis can be spared [25]. Loss of perfusion in cases of suspected testicular rupture is conventionally investigated by CDUS. However, CDUS is considered unreliable in defining ischaemia [6, 7, 25] which could cause delay in appropriate surgical treatment. CEUS can clearly delineate perfused, viable testicular tissues (Fig. 5) increasing the confidence in the diagnosis of testicular rupture and aids in the possibility of testis-sparing surgery [25]. The CEUS appearances of testicular rupture are those of focal or global ischaemia with non-perfused, avascular regions of the testis which breach the tunica albuginea [19]. CEUS can clearly define the interrupted borders of the testis which may be subtle on B-mode imaging [19]. This provides a greater level of diagnostic information than conventional B-mode (Fig. 5) and CDUS and helps to define viability for testis-sparing surgery [19, 25].

While CEUS can increase confidence in the diagnosis of testicular rupture, it is also valuable during the follow-up examination. The prolonged presence of heterogeneity and hypoechoic regions on B-mode imaging may mimic post-surgical infarction or resolving intratesticular haematomas. Clear delineation of vascular perfusion on follow-up imaging can increase the clinician’s confidence in tissue perfusion and avoiding the need for further surgical management.

#### Testicular fracture

Testicular fracture is defined as a traumatic interruption of the testicular parenchyma which may occur independently from disruption of the tunica albuginea [24]. In some cases, secondary rupture of the tunica albuginea may occur due to raised intratesticular pressure secondary to haematoma [1]. Testicular fracture requires rapid diagnosis due to the high rate of orchidectomy associated with the injury [19, 24]. B-mode imaging demonstrates an ill-defined, hypoechoic linear area which traverses the testis [19]; however, visualisation of the fracture plane is only seen in 17% of cases [23].

Testicular fracture vascular assessment is vital for preoperative decision-making. Perfused testicular parenchyma is often viable, and debridement along the fracture line may be performed, salvaging the majority of the testis [23]. CEUS provides more accurate representation, improving assessment of testicular viability and the fracture plane visualisation (Fig. 5). A testicular fracture is seen on CEUS as a clearly marked avascular or hypovascular fracture plane which can be easily differentiated from the adjacent normally perfused testicular parenchyma [1, 19]. Some studies have found that CEUS correctly identified all testicular fractures, with the surgeon benefiting from this ability to detect the precise amount of viable testicular parenchyma in the pre-surgical planning of cases [19]. CEUS can therefore help avoid false negative results in time-critical situations and avoid complications [1].

#### Penetrating testicular trauma

**Fig. 6:**
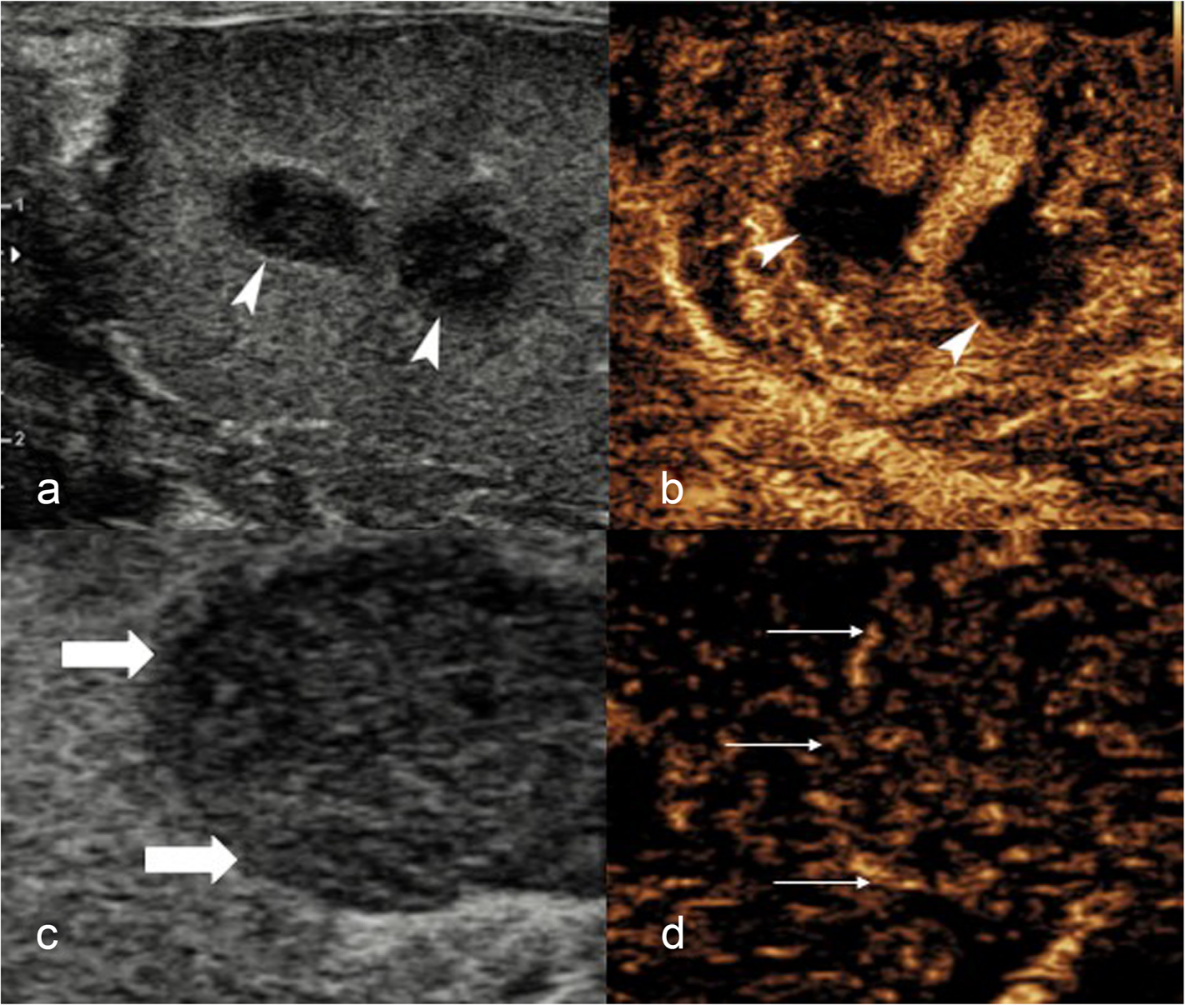
CEUS findings of iatrogenic penetrating testicular injury post-biopsy. B-mode US image (**a**) obtained 24 h following core biopsy of the testis demonstrating two hypoechoic lesions within the central testis (arrowheads), which have typical appearances of intratesticular haematomas. CEUS (**b**) confirmed the avascular nature of the suspected haematomas (arrowheads). A separate patient with a histologically proven testicular tumour. For comparison, (**c**) B-mode shows a well-defined hypoechoic lesion (thick arrow). **d** CEUS demonstrated intrinsic enhancement (thin arrow) in keeping with a testicular tumour, unlike an avascular haematoma

Penetrating scrotal trauma is very rare, and encompasses iatrogenic causes such as testicular biopsy (Fig. 6) through to assault, animal bites and gunshot wounds [23]. Ultrasound is not usually required as many of the cases proceed directly to surgical exploration and debridement [4]. When undertaken, B-mode US may demonstrate the presence of intratesticular haematomas, fractures or haematoceles. However, there may be foreign bodies and obscuring gas within the soft tissues, rendering US examination sub-optimal [4].

The need for sterility in these cases is paramount; the use of sterile gel, transducer covers and thorough transducer cleaning techniques must be followed [26]. Assessment of vascularity in penetrating testicular trauma may be difficult with intrinsic foreign bodies preventing accurate imaging or artefact; however, the advent of CEUS provides a more accurate depiction of global perfusion [6, 7, 25].

A rare consequence of penetrating testicular trauma is a pseudoaneurysm which appears as an anechoic region on B-mode imaging. On CDUS and spectral Doppler US interrogation, oscillating spectral Doppler (“ying yang” appearance on CDUS) is typical of a pseudoaneurysm [23] and can also be seen on CEUS [22].

#### Traumatic haematocele

Presence of blood between the layers of the tunica vaginalis is defined as a haematocele [5, 27] and is the most common scrotal pathology seen following blunt force trauma [23]. Unlike intratesticular haematomas, these occur purely outside the testis as an extra-testicular collection [23]; however, similar to intratesticular haematomas, the US appearance of the haematocele varies depending on the elapsed time from trauma to imaging [23, 27]. Acute haematoceles will demonstrate echogenic appearances; however, chronic haematoceles demonstrate evolutional change such as solid-cystic organisation and septations and temporal decreasing size [23, 27]. In rare cases, there may be a fluid-fluid level seen in old haematoceles, as the blood products settle in gravity-dependent layering [27]. Care must be taken when examining ageing haematoceles as their complex appearances may mimic rare extra testicular neoplasms [28].

**Fig. 7:**
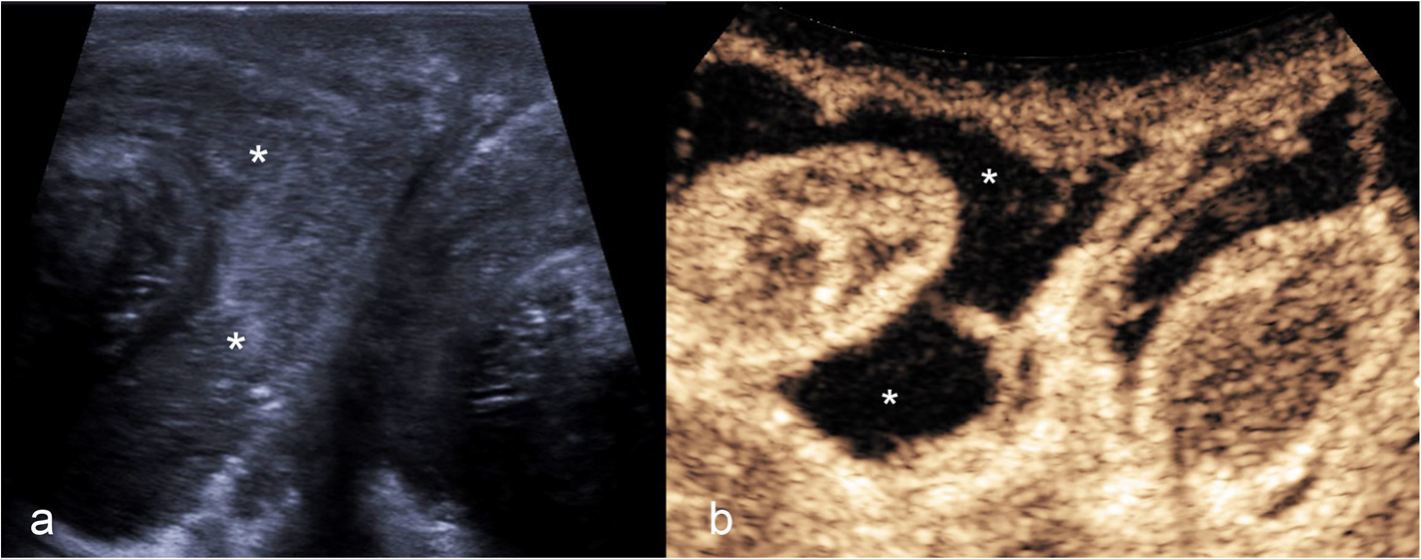
CEUS findings of traumatic haematocele. B-mode US (**a**) showed an echogenic haematocele (asterisks) preventing assessment of the tunica albuginea contour on greyscale imaging. However, CEUS (**b**) clearly demonstrated normal testicular enhancement on both sides, without interruption of the tunica albuginea—excluding testicular infarction or rupture and establishing the diagnosis of a simple haematocele. Note that the haematocele (asterisks) shows no enhancement

In the presence of an acute echogenic haematocele, it is difficult to examine the integrity of the tunica albuginea, limiting the assessment for associated testicular rupture. CDUS assessment of the testis in the setting of surrounding haematocele can be limited, often leading to surgical exploration. However, CEUS offers increased diagnostic yield with regards to identifying parenchymal borders, infarction and tunica albuginea integrity, and differentiating a surrounding haematocoele (Fig. 7) [6, 7, 25].

In cases of a simple haematocele, CEUS will demonstrate uninterrupted tunica albuginea and no enhancement of the haematocele [29]. Furthermore, this helps to differentiate the haematocele from a rare extratesticular neoplasm [6, 7, 1, 19, 28].

#### Extratesticular haematoma

**Fig. 8:**
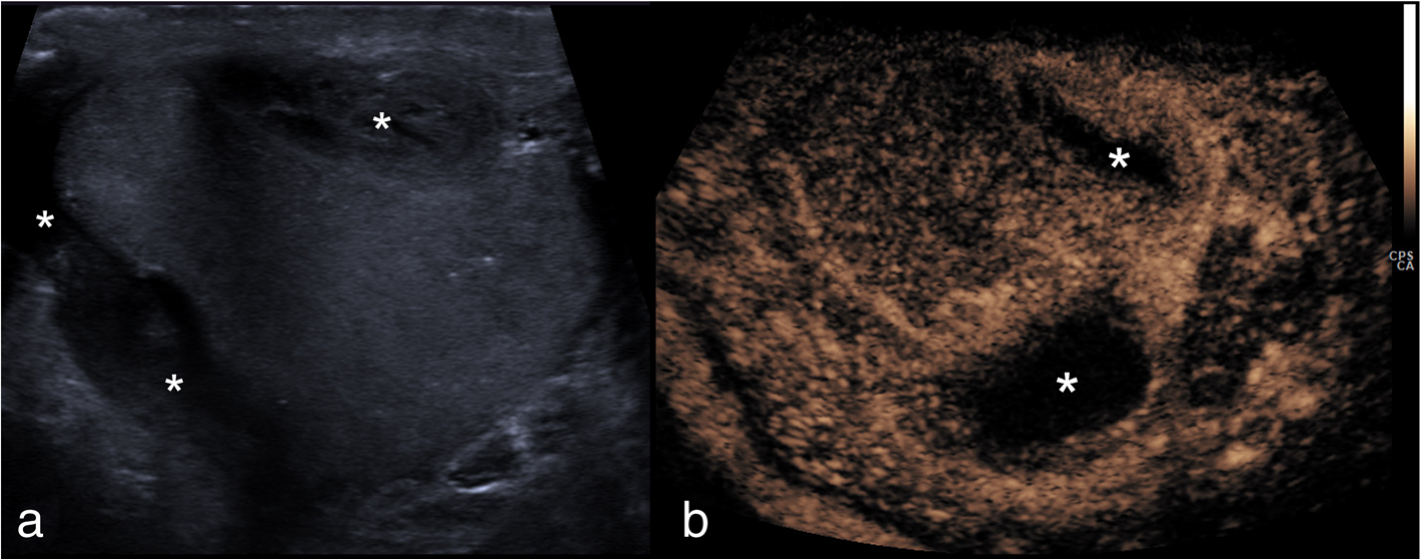
CEUS appearances of extra-testicular haematoma. On B-mode (**a**), a note is made of an extratesticular hypoechoic haematoma (asterisks) causing compression of the testicular parenchyma. Essential issues in such cases include the thorough assessment of testicular parenchymal perfusion and integrity of the adjacent tunica, to rule our testicular rupture. CEUS (**b**) showed uniform enhancement of the testis and excludes focal ischaemia secondary to extrinsic compression, confirming that the tunica albuginea is intact. The extratesticular haematomas shows no internal enhancement (asterisks) but a rim of perilesional hyperaemia

Extratesticular haematomas can originate from a variety of sources from within the scrotum, but also may traverse a patent inguinal canal from the abdomen into the scrotal sac [30]. As a result, abdominal visceral trauma should be considered, particularly in high impact trauma. Large extratesticular haematomas can compromise vascular supply to the testis [30]. Accurate assessment of parenchymal perfusion is essential to detect ischaemia or infarction which may require surgical debridement as opposed to more typical conservative management [1]. Much like the acute haematocele, the presence of adjacent echogenic material to the testis may cause difficulty in evaluation of the tunica albuginea and testicular contour and may also mimic neoplasia [7, 6, 25]. CEUS can confidently confirm the avascular nature of the haematoma and delineate the testicular contour and parenchyma (Fig. 8).

## Conclusions

Whilst the use of CEUS is established in other clinical contexts, use in testicular trauma is less well recognised. CEUS improves the diagnostic accuracy and confidence over conventional B-mode US and CDUS in detecting viable testicular parenchyma after scrotal trauma which is clinically significant in potentiating testis-sparing surgery. CEUS could also play a vital role in helping detect findings which allow conservative treatments and negate unnecessary exploratory surgery or orchidectomy.

## Data Availability

Data sharing is not applicable to this article as no datasets were generated or analysed during the current study.

## Abbreviations

CDUS: Colour Doppler ultrasound
CEUS: Contrast-enhanced ultrasound
CT: Computed tomography
EFSUMB: European Federation of Societies for Ultrasound in Medicine and Biology
FDA: Food and Drug Administration
MI: Mechanical index
MRI: Magnetic resonance imaging
UCA: Ultrasound contrast agent
US: Ultrasound

## References

[1] Lobianco R, Regine R, De Siero M, Catalano O, Caiazzo C, Ragozzino A (2011). Contrast-enhanced sonography in blunt scrotal trauma. J Ultrasound.

[2] Yusuf GT, Sidhu PS (2013). A review of ultrasound imaging in scrotal emergencies. J Ultrasound.

[3] Lee SH, Lee DG, Choi SK, Choi T, Yoo KH (2017). Trends in testicular injury in Korea, 1986-2015. J Korean Med Sci.

[4] Adlan T, Freeman SJ (2014). Can ultrasound help to manage patients with scrotal trauma?. Ultrasound.

[5] Sidhu Paul S. (2011). Diseases of the testis and epididymis. Clinical Ultrasound.

[6] Yusuf G, Konstantatou E, Sellars ME, Huang DY, Sidhu PS (2015). Multiparametric sonography of testicular hematomas: features on grayscale, color Doppler, and contrast-enhanced sonography and strain elastography. J Ultrasound Med.

[7] Jaffer OS, Sidhu PS (2013). Contrast-enhanced ultrasonography of the testes. Ultrasound Clinics.

[8] Food and Drug Administration (FDA) (March 2016) URL: http://www.fda.gov/downloads/drugs/developmentapprovalprocess/ucm071120.pdf.

[9] Sidhu PS, Cantisani V, Dietrich CF (2018). The EFSUMB guidelines and recommendations for the clinical practice of contrast-enhanced ultrasound (CEUS) in non-hepatic applications: update 2017 (short version). Ultraschall Med.

[10] Claudon M, Dietrich CF, Choi BI (2013). Guidelines and good clinical practice recommendations for contrast enhanced ultrasound (CEUS) in the liver - update 2012: a WFUMB-EFSUMB initiative in cooperation with representatives of AFSUMB, AIUM, ASUM, FLAUS and ICUS. Ultrasound Med Biol.

[11] Yusuf GT, Fang C, Huang DY, Sellars ME, Deganello A, Sidhu PS (2018). Endocavitary contrast enhanced ultrasound (CEUS): a novel problem solving technique. Insights Imaging.

[12] Piscaglia F, Nolsoe C, Dietrich CF (2012). The EFSUMB guidelines and recommendations on the clinical practice of contrast enhanced ultrasound (CEUS): update 2011 on non-hepatic applications. Ultraschall Med.

[13] Yusuf Gibran T., Sellars Maria E., Deganello Annamaria, Cosgrove David O., Sidhu Paul S. (2017). Retrospective Analysis of the Safety and Cost Implications of Pediatric Contrast-Enhanced Ultrasound at a Single Center. American Journal of Roentgenology.

[14] Marshall G, Sykes A, Berry J, Jonker L (2011). The “humble” bubble: contrast-enhanced ultrasound. Radiography.

[15] Piscaglia Fabio, Bolondi Luigi (2006). The safety of Sonovue® in abdominal applications: Retrospective analysis of 23188 investigations. Ultrasound in Medicine & Biology.

[16] Yusuf G, Sellars ME, Kooiman GG, Diaz-Cano S, Sidhu PS (2013). Global testicular infarction in the presence of epididymitis: clinical features, appearances on grayscale, color Doppler, and contrast-enhanced sonography, and histologic correlation. J Ultrasound Med.

[17] Lung PFC, Jaffer OS, Sellars ME, Sriprasad S, Kooiman GG, Sidhu PS (2012). Contrast-enhanced ultrasound in the evaluation of focal testicular complications secondary to epididymitis. AJR Am J Roentgenol.

[18] Bertolotto M, Derchi LE, Sidhu PS (2011). Acute segmental testicular infarction at contrast-enhanced ultrasound: early features and changes during follow-up. AJR Am J Roentgenol.

[19] Valentino M, Bertolotto M, Derchi L (2011). Role of contrast enhanced ultrasound in acute scrotal diseases. Eur Radiol.

[20] Seitz K, Strobel D (2016). A Milestone: Approval of CEUS for diagnostic liver imaging in adults and children in the USA. Ultraschall Med.

[21] Greis C (2004). Technology overview: SonoVue (Bracco, Milan). Eur Radiol.

[22] Rafailidis V, Huang DY, Yusuf GT, Sidhu PS (2020). General principles and overview of vascular contrast-enhanced ultrasonography. Ultrasonography.

[23] Bhatt S, Dogra VS (2008). Role of US in testicular and scrotal trauma. Radiographics.

[24] Missiroli Caterina, Mansouri Mohammad, Singh Ajay (2017). Imaging of Acute Conditions of Male Reproductive Organs. Emergency Radiology.

[25] Hedayati V, Sellars ME, Sharma DM, Sidhu PS (2012). Contrast-enhanced ultrasound in testicular trauma: role in directing exploration, debridement and organ salvage. Br J Radiol.

[26] Nyhsen CM, Humphreys H, Koerner RJ (2017). Infection prevention and control in ultrasound - best practice recommendations from the European Society of Radiology Ultrasound Working Group. Insights Imaging.

[27] Deurdulian C, Mittelstaedt CA, Chong WK, Fielding JR (2007). US of acute scrotal trauma: optimal technique, imaging findings, and management. Radiographics.

[28] Barale M, Oderda M, Faletti R (2015). The strange case of a hematocele mistaken for a neoplastic scrotal mass. Can Urol Assoc J.

[29] Sporea I, Badea R, Brisc C (2017). Romanian national guidelines on contrast enhanced ultrasound in clinical practice. Med Ultrason.

[30] Caglayan F, Cakmak M, Karadeniz Y, Atasoy P, Eroglu E, Apan A (2004). Effect of extratesticular hematoma on testicular blood flow and the histology of the testis and dartos fascia. an experimental study. Urol Int.

